# Pediatric migraine and episodic syndromes that may be associated with migraine

**DOI:** 10.1186/s13052-014-0092-4

**Published:** 2014-11-19

**Authors:** Daniele Spiri, Victoria Elisa Rinaldi, Luigi Titomanlio

**Affiliations:** Department of Pediatrics, Luigi Sacco Hospital, Università degli Studi di Milano, Milan, Italy; Department of Pediatrics, Università degli Studi di Perugia, Perugia, Italy; Department of Pediatric Emergency Care, APHP-Hospital Robert Debré, Paris, France; Pediatric Migraine and Neurovascular Diseases Unit, APHP-Hospital Robert Debré, Paris, France; Pediatric Emergency Department, Robert Debré University Hospital, 48, Bld Sérurier, Paris, 75019 France

**Keywords:** Infantile colic, Migraine, Cyclic vomiting, Recurrent abdominal pain, Functional abdominal pain, Torticollis

## Abstract

**Importance:**

Migraine is a common disorder and a frequent cause of medical consultation in children. Many childhood episodic syndromes have been described as common precursors of migraine.

**Objective:**

To review current knowledge on migraine and childhood episodic syndromes, and to discuss future directions for research and clinical practice.

**Findings:**

For most children it is difficult to describe a headache and fully verbalize symptoms such as photophobia and phonophobia that must be inferred from behaviour. Classical migraine features are rare before the age of 6 years, but some migraine-related syndromes have been described. Benign paroxysmal torticollis of infancy, benign paroxysmal vertigo of childhood, cyclic vomiting syndrome and abdominal migraine are currently classified as childhood episodic syndromes, and therefore common precursors of migraine. A strong association between infantile colic and migraine has recently been reported. There are similarities between children with episodic syndromes and children with migraine, regarding social and demographic factors, precipitating and relieving factors, and accompanying gastrointestinal, neurologic, and vasomotor features. The real pathophysiological mechanisms of migraine are not fully understood. Current data obtained through molecular and functional studies provide a complex model in which vascular and neurologic events cooperate in the pathogenesis of migraine attacks. Genetic factors causing disturbances in neuronal ion channels, make a migraineur more sensitive to multiple trigger factors that activate the nociception cascade. The expanding knowledge on migraine genetics and pathophysiology may be applicable to childhood episodic syndromes. Migraine preventive strategies are particularly important in children, and could be beneficial in childhood episodic syndromes. Nonspecific analgesics like ibuprofen and acetaminophen are widely used in pediatrics to control pain and have been found to be effective also in the treatment of acute migraine attacks. Triptans are the specific fist-line drugs for acute migraine treatment.

**Conclusions and relevance:**

Migraine phenotype differs somewhat in the developing brain, and childhood episodic syndromes may arise before typical migraine headache. Diagnosing pediatric migraine may be difficult because of children’s language and cognitive abilities. The risk of underestimating migraine in pediatric age is high. An adequate diagnosis is important to maintain a good quality of life and to avoid inappropriate therapy.

## Background

Headache is the most common pain condition in children and adolescents visiting a pediatrician [1] and migraine is one of the most common causes of primary headache in childhood [2]. Its prevalence increases throughout childhood, affecting 1-3% of 3 to 7 year-olds, 4-11% of 7 to 11 year-olds, and 8-28% of teenagers (13-18 years old) [3]. Migraine is a neurovascular disorder with a genetic background and several genes have been identified as being direct causal or associated agents [4]. First degree relatives of subjects with migraine have a 1.9 times higher risk of developing migraine compared to the general population [5,6] and the concordance rate for migraine with aura in monozygotic twins is 34% compared to 12% in dizygotic twins [7], suggesting the importance of genetic factors in migraine development.

Migraine is disabling at all ages; in children and adolescents it can be accompanied by significant disability, such as school absenteeism, low-quality performance and impaired emotional functioning [8]. Children with recurrent headache can suffer from depression, anxiety and often from sleep disorders and they have the tendency to feel dizzy [9]. Poor performance at school is more likely to be present in children with episodic and chronic migraine, in comparison to children without headaches. School performance is significantly influenced by severity and duration of headache attacks, by abnormal scores of mental health, and by nausea, as well as by headache frequency and use of analgesics [10].

We aimed at reviewing current knowledge and concerns on migraine and related episodic syndromes. To identify articles a literature search was carried out in PubMed and the electronic Science Direct for all studies published until July 31^st^, 2014. The keywords searched for were “migraine and children”, ”headache and children”, and “episodic syndromes and migraine”. Studies were included if they focused on the pediatric population, if they were published in peer reviewed journals and written in English.

### Migraine diagnosis and current state of clinical practice

Headache diagnosis is usually conducted on the basis of the International Headache Society (IHS) criteria. After two editions (respectively in 1988 and in 2004) [11] the latest International Classification of Headache Disorders (ICHD-III beta) is available [12].

### Diagnostic International Headache Society criteria for migraine without aura and migraine with aura

**Migraine without aura**

A. At least 5 attacks fulfilling criteria B-D
B. Headache attacks lasting 4-72 hours (untreated or unsuccessfully treated). In children, attacks may last 2-72 hours.
C. Headache has at least two of the following characteristics:
  1. unilateral location (commonly bilateral in young children)
  2. pulsating quality
  3. moderate or severe pain intensity
  4. aggravation by or causing avoidance of routine physical activity
D. During headache at least one of the following:
  1. nausea and/or vomiting
  2. photophobia and phonophobia
E. Not attributed to another disorder

**Migraine with typical aura**

A. At least 2 attacks fulfilling criteria B-D
B. Aura consisting of at least one of the following, but no motor weakness:
  1. fully reversible visual symptoms including positive features (e.g., flickering lights, spots or lines) and/or negative features (i.e., loss of vision)
  2. fully reversible sensory symptoms including positive features (i.e., pins and needles) and/or negative features (i.e., numbness)
  3. fully reversible dysphasic speech disturbance
C. At least two of the following:
  1. homonymous visual symptoms and/or unilateral sensory symptoms
  2. at least one aura symptom develops gradually over ≥5 minutes and/or different aura symptoms occur in succession over ≥5 minutes
  3. each symptom lasts ≥5 and ≤60 minutes
D. Headache fulfilling criteria B-D for Migraine without aura begins during the aura or follows aura within 60 minutes
E. Not attributed to another disorder

Unlike the previous editions that were mostly based on the opinions of experts, for the latest edition there is substantial evidence available for the classification work. Already in the ICHD-II, criteria for migraine had been specifically adopted for use in children allowing a higher number of pediatric patients to be classified. The two most frequent subtypes of migraine in pediatric population are respectively migraine without aura and migraine with aura. Aura is defined as a transient focal neurological phenomenon that occurs before or during a headache. It appears gradually over several minutes and generally lasts less than 1 hour. Symptoms can be visual, sensory or motor and many children experience more than one symptom.

Migraine is the second most common cause of chronic recurrent headache in school children, with a prevalence ranging from 3.2 to 14.5% [13]. However, epidemiologic studies using ICHD-II demonstrated that in 27-35% of children it was not possible to perform a specific primary headache diagnosis [14-16]. Criteria for migraine diagnosis in children and adolescents are confirmed in ICHD-III beta except for duration of attacks. For the ICHD-II classification migraine may last as little as 1 hour whereas in the new classification, attacks may last from 2 to 72 hours (instead of a minimum of 4 hours in adults): the evidence for untreated migraine with a duration inferior than 2 hours in children has not been substantiated.

The younger the child is, the more atypical the symptoms are. Migraine in preschool children may lack throbbing characteristics, unilaterality and full-blown autonomic symptoms [9] but its paroxysmal and periodic occurrence is observed as the outstanding feature in all migraine manifestations at this age [17]. Children often experience a bilateral pain, usually in frontal or temporal regions, poorly defined and invariably accompanied by pallor, nausea and vomiting. The unilateral pain, typically referred by adults, usually emerges in late adolescence or early adult life. Occipital headache is not common in children, in which case a possible organic pathology should be suspected. Pain quality is different to that of adults because it is often reported as constrictive-oppressive and for most children it is difficult to describe a headache using words like “throbbing” or to fully verbalize symptoms such as photophobia and phonophobia that must be inferred from behaviour [18]. Cranial autonomic symptoms, mainly ocular and nasal (lacrimation, conjunctival injection, nasal congestion, rhinorrhoea), are frequently found in pediatric migraine, often leading to a misdiagnosis of sinus headache and delaying appropriate migraine therapy [19].

In children, migraine with aura is less frequent than migraine without aura. The most common aura symptoms in children are visual and sensorial disorders. Visual aura is typically referred as a “fortification spectrum” characterized by zigzag lines close to the fixation point that then gradually expand assuming the appearance of a convex shape displaced laterally and twinkling. Vision becomes increasingly blurred and children can experience transient hemianopsia or a complete transient unilateral blindness (*amaurosis fugax*). Sensorial aura is often characterized by unilateral paresthesia, hemiparesis and dysphasia. Children typically describe many small pinpricks that slowly move from the place of origin to wider regions as the arm, the leg or the face.

During the last two decades, modifications of ICHD criteria permitted to improve sensitivity of migraine diagnosis in children up to 84.4% [20,21] but studies demonstrate that there still are deficiencies in fully recognizing pediatric headache and Ballottin demonstrated that the ICHD-II criteria are poorly applicable to children below the age of 6 years [22]. Further studies are needed to define the applicability of ICHD-III criteria in the pediatric population and in order to develop alternative and more specific criteria for children. A recent study conducted by De Carlo found that 34.6% of pediatric migraine patients have osmophobia and when this symptom was utilized in the diagnostic process as an additional associated symptom, there was an increase in the percentage of subjects with a migraine diagnosis [23].

### Migraine pathogenetic hypothesis

Eight genes have been so far identified as being associated with common migraine (i.e. with and without aura) [24,25]. *MTDH*, *LRP1*, *PRDM16*, *MEF2D*, *ASTN2*, and *PHACTR1* are involved in neuronal and glutamatergic pathways; *TGFBR* is responsible for the maintenance of vascular integrity and function whereas *TRPM8* is involved in pain signalling pathways [26]. Many advances in the understanding of migraine pathophysiology have been possible through to the study of the rare autosominal dominant Familial hemiplegic migraine (FHM). Gene mutations in membrane ion channel transport proteins, including *CACNA1A* (P/Q-type voltage-gated calcium channel), *ATP1A2* (sodium-potassium ATPase), *SCN1A* (voltage-gated sodium channel), and *PRRT2* (proline-rich transmembrane protein 2) lead to FHM. More recently, mutations in *SLC4A4* [27], encoding the Na^+^-HCO_3_^-^ cotransporter NBCe1, have been identified [28]. Multiple conventional and advanced MRI techniques including susceptibility weighted imaging (SWI) play a key role during an Hemiplegic Migraine attack to exclude acute arterial ischemic stroke; the transient unilateral motor weakness that characterizes this rare type of migraine usually lasts for a few hours and neuroimaging abnormalities completely resolve within 24 hrs after the attack onset [29].

**Figure 1:**
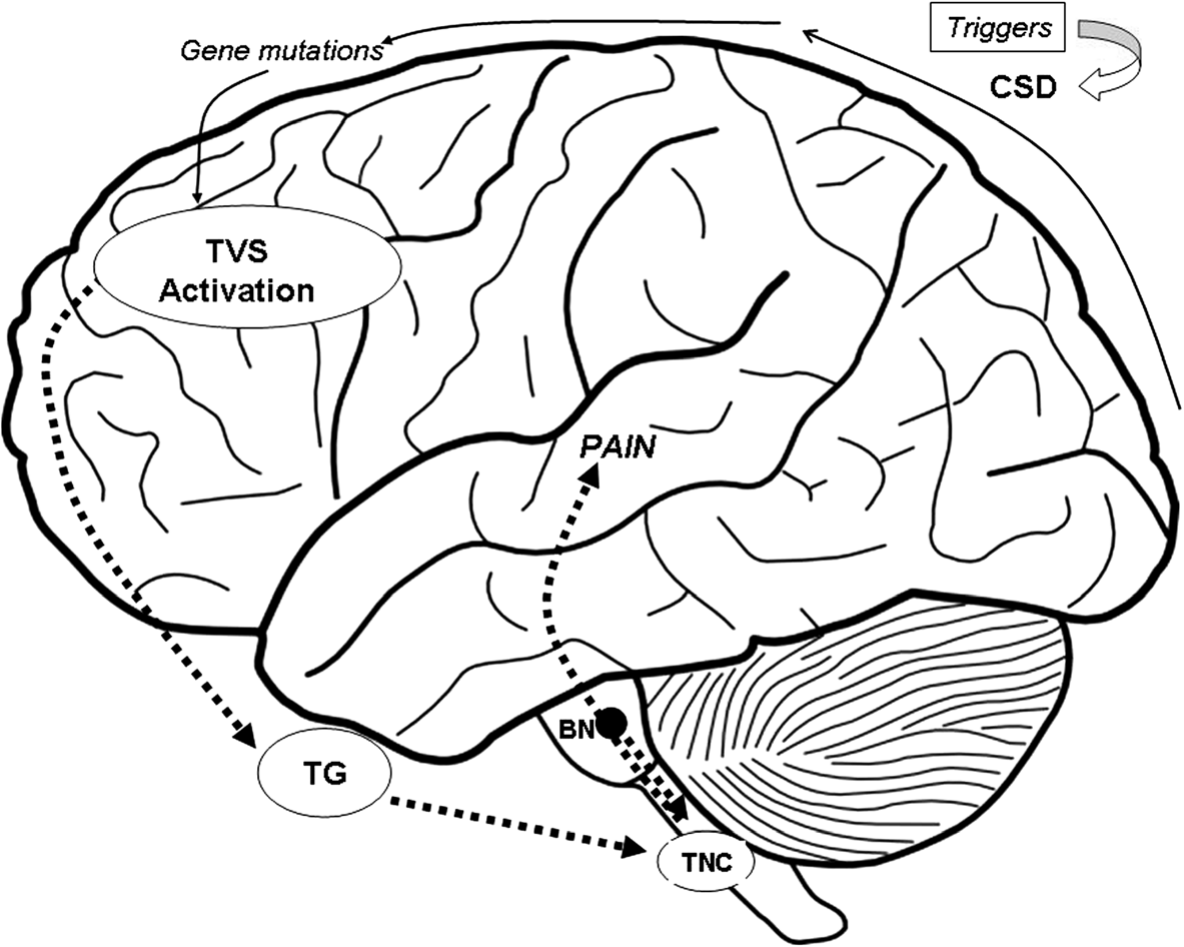
**Migraine pain generation.** Migraine triggers initiate the neuronal excitation that leads to clinical manifestations in children with a genetic vulnerability to migraine. Cortical spreading depression (CSD) triggers plasma protein extravasation from cerebral blood vessels, which in turn activates trigeminal (TG) afferents within the trigemino-vascular system (TVS). Gene mutations could reduce the threshold for firing of TG neurons. Signals are transduced to the trigeminal nucleus caudalis (TNC), which receives modulatory inputs from other brainstem nuclei (BN), such as the periaqueductal gray, the locus coeruleus and the raphe. The TNC projects to rostral brain areas, where the perception of pain is generated.

The real pathophysiological mechanisms of migraine are not fully understood. For decades, the throbbing pain during migraine headache was thought to originate from dilated cranial arteries: unilateral stimulation of extra cranial and intracranial arteries was in fact associated with ipsilateral head pain [30]. During the past two decades the focus on this vascular hypothesis has diminished and neuronal mechanisms have been suggested as generators of migraine headache, without abnormal activation of perivascular sensory fibers [31]. More recently, current molecular and functional studies, conducted using PET, intracarotid SPECT (Single-Photon Emission Computed Tomography), MRA (magnetic resonance angiography), functional MRI and TMS (Transcranial Magnetic Stimulation), allowed to formulate a complex theory in which vascular and neurologic events cooperate in the pathogenesis of migraine attacks without aura [32]. Afferent innervations of intracranial blood vessels, forming the Trigemino-Vascular System (TVS), are the essential substrate for migraine pain in this intricate network [33] (Figure 1). Specific environmental triggers are supposed to initiate the neuronal excitation that leads to clinical manifestations in patients with a genetic vulnerability to migraine. Migraine-specific triggers, as foods containing vasoactive amines (e.g.: tyramine and phenylethylamine), hormonal fluctuation, psychological and environmental triggers as stress, worse mood [34], anxiety, fatigue, sleep deprivation, light, and rapid temperature changes, induce the activation of trigeminal nerve sensory fibers, which innervate intracranial blood vessels, by dilatation of cranial blood vessels. However, Amin in a recent study conducted using MRA during spontaneous unilateral migraine attacks, demonstrates that migraine pain is not accompanied by extracranial arterial dilatation but by only slight intracranial dilatation [35].

Therefore, the initial events that lead to the activation of nociception cascade are still not entirely clear. Brainstem network activation determines the release of vasoactive peptides such as CGRP (calcitonin gene-related peptide) and substance P from trigeminal fibers, inducing vasodilation and neurogenic inflammation [36]. This inflammation increases the trigeminal sensory fibers activation and perpetuates the release of vasoactive peptides (sustaining the transmission of pain impulses to the brain) over a time span of hours to days in correspondence with the duration of a typical migraine episode. The cortical spreading depression (CSD) theory is thought to explain generation of migraine attacks with aura. Transcranial magnetic stimulation and functional MRI studies have shown that at baseline, migraneurs have a state of neuronal hyperexcitability in their cerebral cortex, especially in the occipital region. This suggests that the initial phase of a migraine attack with aura is a wave of cortical spreading depression which is associated with the suppression of spontaneous EEG activity and regional oligemia. CSD begins in the occipital region and moving anteriorly, stops at the central sulcus, and then spreads ventrally to the meningeal trigeminal fibers. Once the CSD occurs, H + and K + diffuse to the pia mater and activate meningeal nociceptors (C-fibers) which release pro-inflammatory agents after TVS activation [37].

Recent studies have suggested that some types of migraine may be related to a mitochondrial dysfunction [38]. Biochemical evidence supporting this relationship consists in the fact that abnormal mitochondrial function determines a high intracellular penetration of calcium, an excessive production of free radicals and a deficient oxidative phosphorylation which ultimately causes energy failure in neurons and astrocytes. These events can trigger migraine mechanisms ultimately causing CSD. Further studies are required to investigate a possible relationship between mtDNA and migraine. Current data therefore provide a complex model in which genetic factors, causing disturbances in neuronal ion channels, make a migraineur more sensitive to multiple trigger factors. The generation of migraine pain is probably a consequence of multiple pathophysiological changes in meningeal tissues, TG (Trigeminal Ganglion), trigeminal brainstem nuclei and descending inhibitory systems that are still not fully clarified and are worth further studies. The result is an activation of the brainstem which is the principal vascular tone regulating and nociceptive processing center. Activation of the brainstem also regulates the nociceptive transition to higher structures of the central nervous system (CNS), as the thalamus and the cortex [39].

### Treatment of migraine

An appropriate migraine management requires filling in a diary for at least one month in order to collect more detailed information on headache characteristics and associated signs.

Migraine preventive strategies are particularly important in children, and could be beneficial in childhood episodic syndromes. Prevention of migraine attacks includes trigger management and adequate pharmacologic treatments, when needed. Frequent migraine triggers, such as physical and mental stress, school problems, irregular and unhealthy lifestyle habits [40] can be removed or modified. A correct lifestyle with appropriate nutritional and sleeping habits and regular physical activity are the first steps to achieve a high quality migraine therapy. Distraction techniques, relaxation and biofeedback are also valuable tools that pediatricians must take into account in managing migraine [41-44].

Acute pharmacological treatment consists in using drugs only during a migraine attack. Its goal is to completely eliminate pain and related symptoms. Nonspecific analgesics like ibuprofen and acetaminophen are widely used in pediatrics in order to control pain and have been studied in randomized, double-blind, placebo-controlled trials and found to be effective also in the treatment of acute migraine attacks [45,46]. Triptans are the specific fist-line drugs for acute migraine treatment [47,48]. Although nasal spray sumatriptan [49-51] and zolmitriptan [52] have positive randomized trials in children, only almotriptan [53] (in adolescents aged 12-17 years) and rizatriptan [54] (for 6-17 years olds) are now FDA-approved for pediatric use. Generally ergot alkaloid dihydroergotamine [55], dopamine receptor antagonists [56] and opioids are not considered first-line treatment of acute migraine in children and adolescents because of their potential side-effects and, especially regarding opioids, because of their association with the development of chronic migraine and medication overuse headache [57,58].

Sheridan et al. [59] recently described the variability in diagnostic testing and treatment of headaches in children presenting to the emergency department and found that despite evidence based clinical guidelines, a large number of children with migraine continued to receive opioids as acute treatment.

Pharmacologic preventive treatment is generally proposed when migraine attacks occur once a week or whenever the attacks are particularly long and debilitating [60]. Among migraine preventives, topiramate [61-63], flunarizine [64], amitriptyline [65,66] and divalproex sodium [67,68] have evidence of efficacy and safety supporting their use in children.

### Current state of clinical practice and diagnosis of childhood episodic syndromes

A range of symptoms and signs (such as vertigo, torticollis, visual and sensorimotor disturbances, anorexia, recurrent abdominal pain, nausea and vomiting, motion sickness, sleep and behavioural disorders) may occur in children in the absence of headache and may precede the development of typical migraine manifestations by several years [69,70].

**Figure 2:**
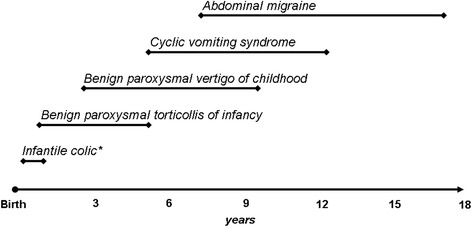
**Age-related expression of childhood episodic syndromes common precursors of migraine.** * = infantile colic is actually considered as an episodic syndrome that may be associated with migraine.

Benign paroxysmal torticollis, benign paroxysmal vertigo, cyclic vomiting syndrome and abdominal migraine are defined as “episodic syndromes that may be associated with migraine” in ICHD-III beta [12]. In 2001, Jan documented a relationship between infantile colic and migraine [71]. His data suggested that children with migraine were more likely to have a personal history of infantile colic and a family history of infantile colic or migraine in any of the first-degree relatives. Children with a history of infantile colic were also more likely to have a positive family history for migraine. Jan supposed that pain and crying in some genetically predisposed infants could represent a form of infantile migraine with an age specific expression. We recently confirmed the association between migraine and infantile colic with a multicenter case-control study [72], suggesting that colic may represent one of the earliest clinical manifestations of migraine such as the episodic syndromes included in ICHD-III beta [18] (Figure 2). Paroxysmal tonic up gaze [73], recurrent abdominal pain [74], motion sickness [75], recurrent limb pain [76], and parasomnias [77] are not clearly associated with migraine and are not yet universally accepted as precursors of migraine.

Childhood episodic syndromes are characterized by reversible and stereotyped attacks, with a periodic occurrence. Children are healthy and neurologically normal between attacks [78-80]. There are strong similarities between children with episodic syndromes and children with migraine, regarding social and demographic factors, precipitating and relieving factors, and accompanying gastrointestinal, neurologic, and vasomotor features [17]. Children with episodic syndromes often have a positive family history for migraine and those with earlier-onset episodic syndromes may eventually develop migraine in adolescence or adult age [81]. The clinical improvement observed with migraine-specific drugs (triptans) further confirms the association of episodic syndromes with migraine. Diagnosis of childhood episodic syndrome is one of exclusion and involves a careful anamnesis, physical examination and appropriate neurodiagnostic studies to exclude other discernable causes as epilepsy, metabolic disorders, ischemic events or psychological disorders.

#### Infant colic

Recent research suggests that infantile colic may be a childhood episodic syndrome [72,80]. Infantile colic affects 5-19% of babies during their first months of life and, according to Wessel criteria, it is characterized by inconsolable crying and fussing for more than 3 hours per day, more than 3 days per week and for more than 3 weeks in an otherwise healthy and well-fed infant [82-84].

#### Benign paroxysmal torticollis of infancy (ICHD III beta 1.6.3)

Benign paroxysmal torticollis is a rare paroxismal dyskinesia that appears commonly around the age of 2 to 8 months. It is characterized by recurrent and stereotyped attacks of abnormal inclination or rotation of the head, occasionally accompanied by vomiting and ataxia. Torticollis is secondary to cervical dystonia although it may occur in association with other dystonic features such as truncal and pelvic asymmetrical posturing [78]. During an episode it is possible to observe pallor, photophobia, ataxia, drowsiness and headache which resemble migraine features. The episodes often last from several hours to days, and have a spontaneous resolution. Typically, the attacks frequency and duration decline as the child grows older with a definitive resolution at the age of 5 years [85].

#### Benign paroxysmal vertigo of childhood (ICHD III beta 1.6.2)

Benign paroxysmal vertigo occurs as a sudden attack of unexplained fright accompanied by balance troubles or even falls [86]. The onset is characterized by a sudden expression of anxiety and fear followed by attempting to grasp a person or any other support standing nearby. Autonomic signs such as dizziness, nausea, pallor, perspiration, photophobia and phonophobia may accompany vertigo. Vomiting and nystagmus are common. The episodes, lasting generally less than 5 minutes, are never associated with loss of consciousness [87]. The onset is between the age of 2 to 4 years and the frequency of attacks varies from once a day to once every 1-3 months [88]. Typically, children suffering from benign paroxysmal vertigo have a positive family history for migraine and a positive family and personal history for motion sickness. Some patients may develop other childhood episodic syndromes such as cyclic vomiting or recurrent abdominal pain [89]. Some authors suggest that benign paroxysmal vertigo may constitute an early-onset variant of basilar-type migraine [90].

#### Cyclic vomiting syndrome (ICHD III beta 1.6.1.1)

This syndrome is characterized by recurrent and self-limited episodes of severe nausea and vomiting, interspersed with symptom-free periods. Affected children usually experience a stereotypical pattern of events in which a prodromal phase is followed by an emetic and a recovery phase [91]. During the prodromal phase, lasting about 1.5 hours, children experience an impending episode characterized by worsening nausea and a dramatic autonomic dysfunction with decreased muscle tone, pallor, lethargy and apathy. This clinical picture evolves into emetic phase in which vomiting is typically intense, often bilious, and accompanied by persistent nausea, anorexia, retching, increased salivation, abdominal pain, headache, pallor, photophobia and phonophobia. This phase lasts an average of 24 hours before the beginning of the recovery phase characterized by remission of nausea and resumption of normal appetite, of oral intake and of a baseline clinical status [92].

The onset of cyclic vomiting syndrome occurs generally before the age of 6 years and the frequency of episodes varies between 3 and 12 per year [93]. The median age for resolution of vomiting episodes is 10 years and 75% of affected children will develop migraine by the age of 18 years [94].

#### Abdominal migraine (ICHD III beta 1.6.1.2)

Abdominal migraine is characterized by recurrent, acute-onset abdominal pain lasting for hours or days and accompanied by dysautonomic signs such as pallor, dark shadows under the eyes, flushing, anorexia and vomiting [95]. Affected children experience a dull or colicky pain generally localized in periumbelical region. During the inter-episodic period children are completely healthy [96]. The onset of abdominal migraine occurs at the age of 7 years with a peak of prevalence at 10 years of age [97]. Some studies suggest that abdominal migraine could persist into adult life and 70% of affected children develop migraine as they grow older. There are similarities between affected children and children with migraine regarding demographic, social, clinical and familial features. Moreover, children with abdominal migraine manifest similar triggering events such as psychological stress, physical exhaustion and motion sickness [93]. Sometimes, a preceding aura occurs with visual disturbance, flashing lights, numbness or a tingling sensation, slurred speech or muscle weakness.

### Childhood episodic syndromes pathogenetic hypothesis

The expanding knowledge on migraine genetics and pathophysiology may be applicable to childhood episodic syndromes. According to the fact that both gut and nervous system are derived from the same embryologic tissues and that the enteric nervous system and the CNS exert direct effects on each other [98], it has been hypothesized that pathogenesis of abdominal migraine consists in an increased arousal in the CNS in response to triggers, thus releasing neuropeptides and neurotrasmitters that lead to dysregulation of the gastrointestinal system. This could also explain the recent demonstrated association between infantile colic and migraine. We supposed that infants with colic experience a sensitization of the perivascular nerve terminals in the gut, as happens in the brain during a migraine attack [72]. The potential role of molecules as CGRP, involved in modulation of sensory activity in both the brain and the gut, remains to be elucidated [99]. A disrupted sleep pattern might be a trigger for colic symptoms, as it is often observed in patients with migraine [100]. Cyclic vomiting syndrome is also believed to be a brain-gut disorder involving neuroendocrine pathways in genetically predisposed individuals [101,102]. The same dysregulated pathways may be involved in symptoms accompanying migraine attacks as nausea and vomiting [103].

Mutations in the CACNA1A and PRRT2 genes, which are associated with FHM, have been found in some patients with benign paroxysmal torticollis and benign paroxysmal vertigo [85,104], thus confirming their link to migraine. Transitory vascular disturbances in cerebellar cortex, where CACNA1A is abundantly expressed, were demonstrated in temporal cortex and the vestibular nuclei respectively during episodes of torticollis and vertigo [105].

### Treatment of episodic syndromes

No specific treatment exists for infant colic. Positive results have been obtained by decreasing the infant’s level of stimulation during acute episodes [106,67] as it is observed in migraine. There are no known effective treatments for both benign paroxysmal torticollis and vertigo. Antiemetics could be considered in the acute phase if there is significant vomiting. Acute treatment of cyclic vomiting syndrome typically consists in rehydration and antiemetics [18]. A favourable response to subcutaneous and nasal spray sumatriptan has been reported [68] and represents another argument in favour of a relationship with migraine. Abdominal migraine generally responds to triptans [106]. Preventive treatment with flunarizine can be considered if attacks are frequent or long-lasting [107].

### Remaining concerns and future directions for migraine and related syndromes

ICHD criteria are useful to simplify and standardize diagnostic approach to primary headache, and represent the gold standard for diagnosing migraine and childhood episodic syndromes. However, applying diagnostic criteria to children can be difficult because the course of the disease and the description of the acute events are often inaccurate, especially regarding parameters like duration, intensity and localization [108]. The ICHD-II criteria (and probably the new ICHD-III beta criteria too) are still poorly applicable to children under the age of 6 years [22] and it is likely that infantile colic and osmophobia will be considered respectively as a precursor and an alternative symptom of migraine in future revisions, because of the strong association observed. Modification of the criteria in order to include bilateral headache, shorter duration of the attacks, nausea and/or vomiting associated with at least two out of five other associated symptoms (photophobia, phonophobia, difficulty in thinking, light-headedness or fatigue) which must be derived form behavior, in addition to the usual description of moderate to severe pain of a throbbing or pulsating nature, worsening or limiting physical activity, improved accuracy of migraine diagnosis in children [20,22]. Indeed, when migraine is under-diagnosed the risk is of an immoderate use of analgesics: it is demonstrated that 42.5% of subjects with headache use medicines. 68.2% of these reported that medicines for headache were always available at home, and 22.2% were allowed to use these without asking for permission [109]. Since drug overuse makes treatment of headache difficult to deal, it is important to supervise this condition, which is particularly common in adolescents [110].

Significant progresses in molecular genetics have advanced our understanding of the genetic basis of migraine and related syndromes, also revealing promising treatment targets for future drug development. Genetic findings have revealed ion channels and transporter mutations as causative of migraine [111], which seems to be related to ionic disturbances with a resultant altered excitability of certain areas of the brain and an age-specific phenotype.

New technological strategies such as next-generation sequencing, may aid in the identification of FHM related genes and promote the research for the missing heritability of common migraine [112]. These platforms are able to generate more sequence data; they are less expensive and could help us in finding answers to how migraine attacks start [113] and what the links with childhood episodic syndromes are. It is likely that the study of epigenetic mechanisms (i.e. DNA methylation and post-translational modifications of histone proteins), which affect gene expression and cellular responses to environmental signals, may explain in the next future how non-genetic triggers may modulate attack frequency of migraine [114]. It is realistic to imagine that further studies may reveal tractable drug targets, and this will also help in the understanding of migraine pathogenic process, which is essential for a rational development of effective treatments.

Development of primary headache may be furthered by environmental conditions [115-117]. A child’s environment includes home, school, and community and all of these areas may have profound influences on headache [118]. An association between migraine, parental-reported emotional problems and self-reported anxiety suggests that emotional factors may be early contributors in the pathogenesis of pediatric headache [119,120].

Children with frequents attacks of paroxysmal vertigo or paroxysmal torticollis are very limited in daytime activities and this also influences familial organisation. The same is true for cyclic vomiting attacks, during which children are often hospitalized for intravenous rehydration, and for abdominal migraine, which attacks are often debilitating and can last for days. When migraine attacks are too frequent, too long, or too painful, children’s quality of life can be affected. Therefore, it could be useful to assess the level of life quality by validated scores [121], not only in migraine but also in episodic syndromes, when possible. Performing a score during the initial diagnostic assessment can also help in monitoring the therapeutic response: changes in score are an index of the adequacy of undertaken treatment. Several studies have helped to define the relationship between psychiatric and psychological co morbidities in children suffering from migraine and cyclic vomiting. Whether these comorbidities are triggers or the consequence of a chronic invalidating condition should be proven further.

## Conclusions

Pediatricians are frequently involved in the care of children and adolescents with headache.

Diagnosing pediatric migraine may be difficult because of children’s language and cognitive abilities and it is frequently necessary to detect migrainous symptoms from behaviour. The risk of underestimating the real prevalence of migraine in pediatric age is high and an adequate diagnosis is important to limit uncomfortable situations in school, social and family settings, to maintain a good quality of life and to avoid inappropriate therapy.

Migraine phenotype differs somewhat in the developing brain, and childhood episodic syndromes may arise before typical migraine headache. Being familiar with the characteristics and evolution of childhood episodic syndromes may help pediatricians make correct diagnosis and give adequate treatments, avoiding unnecessary investigations.

## Abbreviations

CGRP: Calcitonin-gene-related peptide
CNS: Central nervous system
CSD: Cortical spreading depression
EEG: Electroencephalogram
FDA: Food and Drugs Administration
FHM: Familial hemiplegic migraine
ICHD: Criteria of the International Classification of Headache Disorders
MRI: Magnetic resonance imaging
PET: Positron emission tomography
SPECT: Single-photon emission computed tomography
MRA: Magnetic resonance angiography
TG: Trigeminal Ganglion
TMS: Transcranial magnetic stimulation
TVS: Trigemino-vascular system
